# Baicalin reduces blood lipids and inflammation in patients with coronary artery disease and rheumatoid arthritis: a randomized, double-blind, placebo-controlled trial

**DOI:** 10.1186/s12944-018-0797-2

**Published:** 2018-06-23

**Authors:** Yuanxing Hang, Xian Qin, Tianli Ren, Jianing Cao

**Affiliations:** Wuxi No.2 People’s Hospital, 68 Zhongshan Road, Wuxi, 214000 Jiangsu China

**Keywords:** Baicalin, Coronary artery disease, Rheumatoid arthritis, Inflammation, Lipids

## Abstract

**Background:**

Patients with rheumatoid arthritis (RA) have an increased risk of coronary artery disease (CAD) above the baseline. Baicalin possesses beneficial effects against both RA and CAD, but little is know on its clincial efficacy among patients manifesting both CAD and RA.

**Methods:**

Three hundred seventy four patients with CAD and RA were randomized to receive either 500 mg baicalin or placebo orally everyday for 12 weeks. Lipid profile, cardiotrophin-1 (CT-1), high sensitivity C-reactive protein (hs-CRP), European League Against Rheumatism (EULAR) response were analyzed at the end of study period.

**Results:**

After 12 week treatment, levels of triglycerides, total cholesterol, LDL-cholesterol and apolipoproteins, as well as CT-1 and hs-CRP, were all significantly improved in the baicalin group compared to the placebo group (1.12 ± 0.36 vs 1.87 ± 0.46 mmol/L, 2.87 ± 1.23 vs 3.22 ± 1.07 mmol/L, 1.38 ± 0.41 vs 1.16 ± 0.32 mmol/L, 1.31 ± 0.41 vs 1.23 ± 0.29 g/L, 42.9 ± 13.7 vs 128.4 ± 24.3 ng/mL, 1.64 ± 0.38 vs 3.9 ± 1.4 mg/dL, respectively). Significantly higher proportion of patients in the baicalin group (71%) reported good/moderate EULAR response than the placebo group (53%).

**Conclusion:**

Baicalin reduces blood lipids and inflammation in patients with both CAD and RA, supporting its further clinical application.

## Background

Rheumatoid arthritis (RA) is an inflammatory auto-immune disease that combines joint swelling and tenderness with synovial joint degradation [2]. RA patients are at a higher risk of mortality than the general population [16, 29], particularly because of their elevated risk of coronary artery disease (CAD) [12]. Accelerated coronary artery atherosclerosis is reported to directly contribute to increased incidences of CAD and even death [5, 9, 16]. Although common risk factors can contribute to CAD incidences in RA patients and the general population, they can not entirely account for the increased CAD events reported in RA patients [7, 18, 25]. A major factor contributing to increased CAD and cardiovascular events in RA patients is probably the elevated systemic inflammation level which are a characteristic RA symptom [26]. In particular, RA-associated inflammation increases risks of CAD by compromising vessel wall integrity, as well as through adversely regulating common risk factors of CAD [23, 28]. Moreover, RA patients in general have marginally higher risk of CAD becaused of their lower levels of cardiorespiratory fitness (CRF) than non-RA individuals [24]. Therefore, therapeutic agents that target both common CAD risk factors and systemic inflammation are urgently needed to alleviate CAD risks among RA patients.

Baicalin is a major bioactive ingredient of the traditional Chinese herbal medicine (CHM) *Scutellaria baicalensis Georgi*. This naturally existing flavone exhibits various pharmacological activity and high clinical value, including anti-inflammatory effects [8]. Baicalin showed specific inhibition against collagenase reaction [19]. In arthritic mouse model, baicalin administration significantly reduced ankle swelling [35]. In addition, in collagen-induced arthritic (CIA) rats, baicalin treatment also relieved joint inflammation [33]. These studies have suggested that baicalin might be a promising novel therapeutic agent for treating RA in humans. On the other hand, baicalin has also exhibited beneficial effects against CAD. For instance, in a chronic pressure-overload mouse model, baicalin suppressed cardiac hypertrophy and fibrosis, thereby attenuating pressure-overload-induced cardiac dysfunction and ventricular remodeling [36]. Moreover, in rats with renovascular hypertension, baicalin reduced pathological changes in the myocardium and myocardial apoptosis, thereby reverting left ventricular remodeling [6]. Baicalin has also been used as anti-arrhythmic and anti-hypertensive drugs in clinical application currently [14, 15].

However, to date, no study has been performed on the effects of baicalin on CAD in RA patients. In the current study, we aimed to investigate the therapeutic effect of oral baicalin administration among patients with both CAD and RA.

## Methods

### Patients

In this intention-to-treat study, 374 patients with CAD and RA were recruited at Wuxi No.2 People’s Hospital from May 2014 to May 2017. The study was approved by the Ethical Committee of Wuxi No.2 People’s Hospital. All participants signed informed consent forms and agreed to our anonymous data utilization policy.

### Inclusion and exclusion criteria

Inclusion criteria were as follows: 1) 45 years of age or older; 2) diagnosed with RA according to the American College of Rheumatology (ACR) criteria [2]; 3) receiving ≥3 months of unchanged antirheumatic drugs and ≥ 1 month of unchanged non-steroidal anti-inflammatory drugs; 4) having moderate or high risk of CAD. A total of 374 patients were initially included in the study.

Exclusion criteria were as follows: 1) having known cerebral, coronary or peripheral artery disease; 2) having one or more arthroplasties of weight bearing joints; 3) receiving ≥3 months of baicalin supplement prior to joining the study. CAD risk was determined based on age and the presence of CAD risk factors (smoking, hypertension, obesity, diabetes, etc). For the purpose of this study, males 45 years of age or older or males younger than 45 years with two or more CVD risk factors are considered to be at moderate risk of CVD [10]. Females 55 years of age or older or females younger than 55 years with two or more CAD risk factors are considered to be at moderate risk of CVD [10]. Only individuals who have signs or symptoms of CAD or have been diagnosed with CAD are considered at high risk. High-risk CAD individuals are only eligible to participate in the study if they have not been prescribed statin for any reason. A total of 23 patients were excluded.

### Study design

The remaining 351 eligible patients were then randomized by a permuted-block design stratified to their baseline EULAR into two treatment groups: 1) baicalin group (*n* = 175), who were orally administered 500 mg baicalin on a daily basis for a period of 12 weeks; 2) placebo group (*n* = 176), who were orally administered placebo on a daily basis for 12 weeks. Both baicalin and placebo were provided to all patients in closed labels to make the contents blind to both the patients and the investigators. All patients were also prescribed 20 mg atorvastatin via oral administration once daily, and 8 mg/kg tocilizumab i.v. every 4 weeks. All patients were instructed not to consume any medication containing baicalin during the 12 week study period. All patients were visited on a monthly basis to ensure compliance to the study protocol. During the 12 weeks study period, 9 patients in the baicalin group and 11 patients in the placebo group, respectively, were withdrawn from the study due to non-compliance or personal reasons, whose data were excluded from the final analysis.

### Evalustions and endpoints

Fasting peripheral venous blood was collected in the morning before treatment and after 12 weeks of treatment for evaluations of CAD parameters. Levels of total cholesterol (TC), triglycerides (TG), low-density lipoprotein cholesterol (LDL-C), high-density lipoprotein cholesterol (HDL-C), apoprotein A_1_ (ApoA_1_) and B_100_ (ApoB_100_), high sensitivity-C reactive protein (hs-CRP) and cardiotrophin-1 (CT-1) were detected using DXI800 and AU5800 automatic biochemical analyzers (Beckman, United States of America). For RA parameters, the endpoint was defined as the proportion of patients maintaining good/moderate European League Against Rheumatism (EULAR) response, according to previously published standard [1].

### Statistial analysis

Statistical analysis was conducted using the SPSS software (SPSS Inc., USA). Data were presented as mean ± standard deviation (SD). The data distribution normality was determined using Kolmogorov-Smirnov goodness-of-fit test. A two-tailed student t-test was employed to calculate significance of normally distributed data, while Mann-Whitney test was employed to calculate significance of non-normally distributed data. *P* values less than 0.05 were regarded as statistically significant.

## Results

**Fig. 1:**
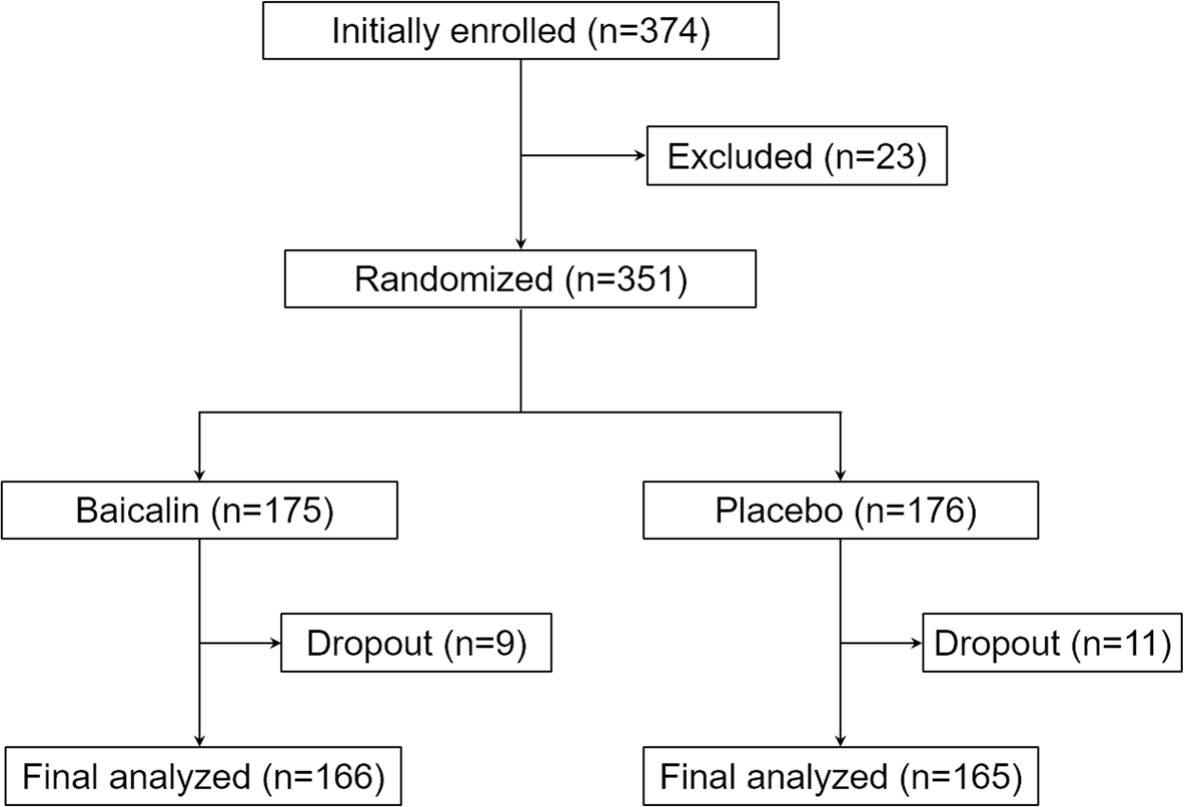
Randomized study design

This was an intention-to-treat study, and the randomized study design is shown in Fig. 1. A total of 374 patients were initial recruited into the study, in which 23 were excluded. The remaining 351 patients were then randomized into either baicalin or placebo treatment groups. During the 12 weeks study period, 9 patients in the baicalin group and 11 patients in the placebo group, respectively, were withdrawn from the study due to non-compliance or personal reasons, whose data were excluded from the analysis. Finally, data from 166 eligible patients in the baicalin group and 165 eligible patients in the placebo group, respectively, were analyzed. As shown in Table 1, baseline characteristics of all eligible patients from the two treatment groups were listed and compared. We didn’t observe any significant differences between the two groups in terms of age, gender, body mass index, smoking, blood pressures and history of RA diagnosis.

**Table 1:** Baseline characteristics of eligible patients

	Baicalin (*n* = 166)	Placebo (*n* = 165)	*P* value
Age (years)	51.3 ± 4.2	52.1 ± 5.8	> 0.05
Gender (male/female)	74/92	67/98	> 0.05
BMI (kg/m^2^)	25.9 ± 4.1	26.3 ± 3.7	> 0.05
Smoking (n)	49	57	> 0.05
SBP (mmHg)	143.7 ± 7.6	148.2 ± 6.3	> 0.05
DBP (mmHg)	86.1 ± 8.3	89.4 ± 7.8	> 0.05
Time of RA diagnosis (years)	5.1 ± 2.4	5.8 ± 2.9	> 0.05

Values are mean ± SD. *BMI* body mass index, *SBP* systolic blood pressure, *DBP* diastolic blood pressure

Next, we assessed the lipid profile of eligible patients from the two groups (Table 2). In both groups, TG, TC, HDL-C, LDL-C, ApoA_1_ and ApoB_100_ were all significantly improved after the common treatment of 20 mg atorvastatin via oral administration once daily and 8 mg/kg tocilizumab i.v. every 4 weeks. Importantly, comparing between the two groups post treatment, all the above parameters were also improved to a better extent in the baicalin group than the placebo group. Similary for CT-1 and hs-CRP levels, marked improvements were also observed in both groups of eligible patients (Table 3). Moreover, the extent of improvements in the baicalin group was also significantly better than those in the placebo group.

**Table 2:** Changes in lipid profile of eligible patients before and after treatment

	Baicalin (*n* = 166)		Placebo (*n* = 165)	
	Baseline	Post treatment	Baseline	Post treatment
TG (mmol/L)	2.77 ± 1.26	1.12 ± 0.36 *	2.83 ± 1.35	1.87 ± 0.46 *#
TC (mmol/L)	4.62 ± 1.53	2.87 ± 1.23 *	4.75 ± 1.62	3.22 ± 1.07 *#
HDL-C (mmol/L)	0.96 ± 0.35	1.38 ± 0.41 *	1.02 ± 0.24	1.16 ± 0.32 *#
LDL-C (mmol/L)	2.84 ± 0.71	1.73 ± 0.52 *	2.91 ± 0.83	2.42 ± 0.57 *#
ApoA_1_ (g/L)	1.13 ± 0.22	1.31 ± 0.41 *	1.16 ± 0.27	1.23 ± 0.29 *#
ApoB_100_ (g/L)	0.83 ± 0.21	0.43 ± 0.11 *	0.86 ± 0.25	0.67 ± 0.23 *#

Values are mean ± SD. *p < 0.05 compared to baseline within the same group; #*p* < 0.05 compared at the same time point between two groups. *TG* triglycerides, *TC* total cholesterol, *HDL-C* high-density lipoprotein cholesterol, *LDL-C* low-density lipoprotein cholesterol, *ApoA*_*1*_, apolipoprotein A_1_, *ApoB*_*100*_ apolipoprotein B_100_

**Table 3:** Changes in CT-1 and hs-CRP levels of eligible patients before and after treatment

	Baicalin (*n* = 166)		Placebo (*n* = 165)	
	Baseline	Post treatment	Baseline	Post treatment
CT-1	194.6 ± 42.3	42.9 ± 13.7 *	187.4 ± 39.1	128.4 ± 24.3 *#
hs-CRP	32.4 ± 2.3	1.64 ± 0.38 *	30.8 ± 2.7	3.9 ± 1.4 *#

Values are mean ± SD. *p < 0.05 compared to baseline within the same group; #*p* < 0.05 compared at the same time point between two groups. *CT-1* cardiotrophin-1, *hs-CRP* high-sensitivity C-reactive protein

**Fig. 2:**
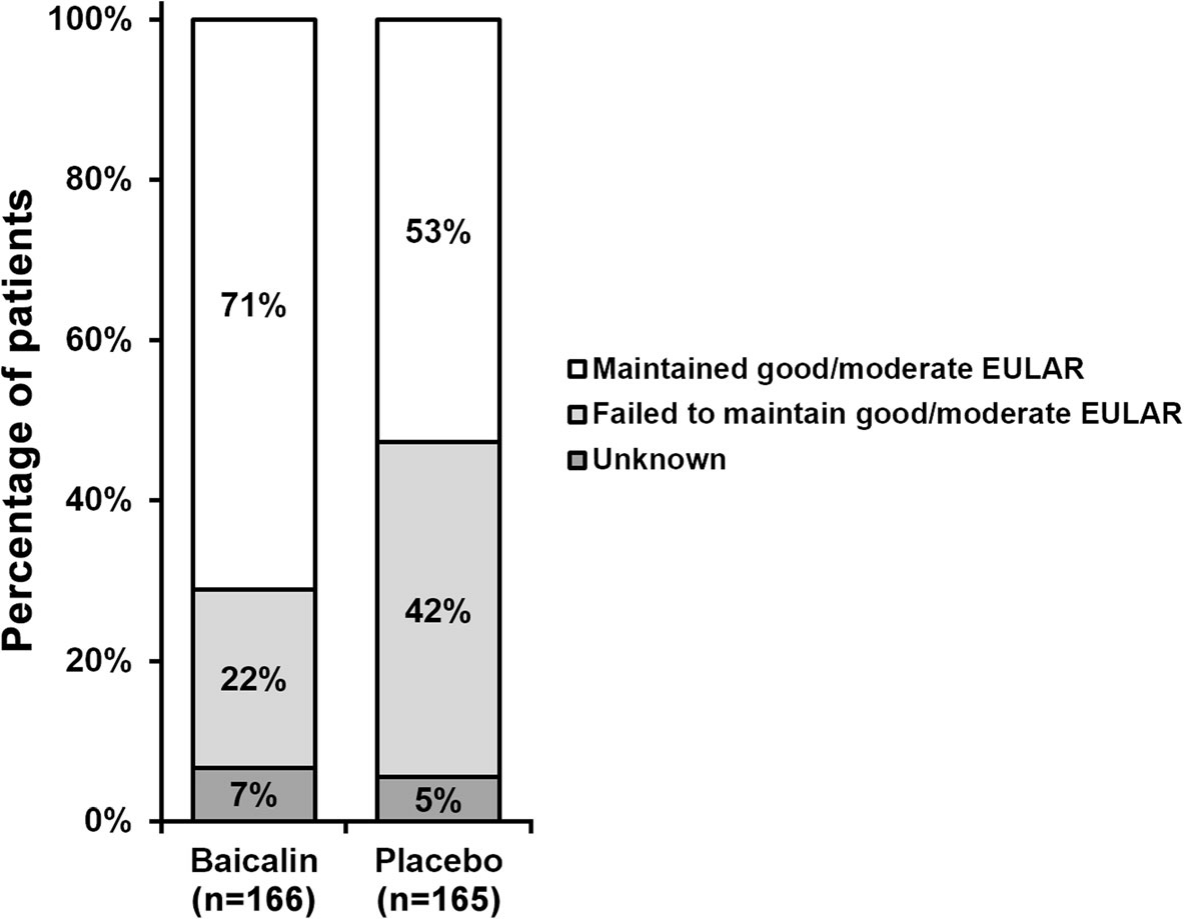
Proportion of eligible patients maintaining good/moderate EULAR response after treatment

EULAR is an evalutaion standard for RA patients [1], which was used in the current study to assess the efficacy of baicalin against RA symptoms. As shown in Fig. 2, the proportion of patients who managed to maitain good/moderate EULAR in the baicalin group was significantly higher than that in the placebo group. On the other hand, percentage of patients who failed to maintain good/moderate EULAR in baicalin group was also lower than in the placebo group.

## Discussion

RA patients are exposed to higher risk of CAD, partially becaused of their elevated chronic systemic inflammation levels as a result of their RA conditions [26]. RA-induced inflammation was reported to accelerate progression of plaques in the vasculature [23, 28]. RA patients typically have lower CRF, which likely further potentiates their risk of CAD [24]. Therefore, there is an urgent need to lower CAD risk among RA patients. Dysregulated metabolism of lipids is a major risk factor contributing to CAD. In line with this, therapies that aim to reduce lipid content is important in preventing and treating CAD [4, 13]. To date, statins are the majorly accepted lipid-lowering therapeutic agents [3, 20, 30]. However, most lipid-lowering investigations aiming at better clinical efficacy generally achieve their goal using high statin doses, which have led to higher incidence rate of adverse effects [17]. Besides statin, nutraceuticals have also been reported to successfully combat dyslipidaemia when statins and other drugs fail in lipid levels reduction [27].

Chinese herbal medicine (CHM) has been deemed as complementary therapy of modern Western medicine [11]. In fact, CHM has a long history and is widely used in the treatment and prevention of various diseases worldwide. Bioactive ingredients of CHM have been reported to reduce infarct volume, improve neurological function and promote endogenous neurogenesis after ischemic stroke [21].

In the context of the current study, we aimed to investigate the therapeutic effects of oral baicalin administration among patients with both CAD and RA. After 12 weeks, we found that lipid profiles of CAD and RA patients were significantly improved by 20 mg atorvastatin via oral administration once daily and 8 mg/kg tocilizumab i.v. every 4 weeks, confirming the effectiveness of this common treatment. Importantly, we observed better improvements in patients treated by baicalin than those on placebo treatment, demonstrating the beneficial effect of baicalin on further improving lipid profile. Moreover, the extent of improvements with regard to CT-1 and hs-CRP, two important risk factors of CAD, was also significantly better in the baicalin group than that in the placebo group, further indicating the efficacy of baicalin in alleviating CAD risks. In terms of RA symptom evaluation, we employed the EULAR as the indicator of RA in the present study. It was observed that proportion of patients maitaining good/moderate EULAR in baicalin group was significantly higher than that of placebo group, while percentage of patients failing to maintain good/moderate EULAR in baicalin group was also lower than in the placebo group. These data clearly supported that, besides lowering CAD risks, baicalin also exhibited potent efficacy in attenuating RA symptoms.

Granted that baicalin possesses dual benefical effects against both CAD and RA, yet the underlying molecular mechanism is unclear. Baicalin was reported to reverse the effect of myocardial injury and inflammation in a mouse model of myocardial ischaemic injury [34]. The authors found that administration of baicalin was able to reduce hs-CRP [34], which was consistent with results in our current study. Furthermore in the same study, baicalin was also shown to suppress inflammation by blocking the aryl hydrocarbon receptor (AhR) [34]. Increasing evidences to date have pointed to an importance for AhR in controlling the extent of inflammatory reactions in response to heart diseases in animal models [22, 31, 32], and it would be interesting to test whether AhR was also affected by baicalin in clinical settings.

In the context of RA, baicalin administration significantly reduced ankle swelling in an arthritic mouse model [35]. In the same study, baicalin treatment also inhibited splenic Th17 cell population expansion. Investigations into the underlying molecular mechanism have indicated that, baicalin could inhibit expression of RORgt gene, a key transcription factor in the differentiation of Th17 cells. Baicalin treatment in an IL-17-contaminated synoviocytes culture greatly suppressed the adhesion of lymphocytes to synoviocytes, and inhibited the inflammatory cascade following IL-17 induction, as well as downregulated expressions of pro-inflammatory cytokines [35]. In addition, baicalin could alleviate joint inflammation in CIA rats by suppressing synovial expression of NF-κB p65 subunit [33]. Future studies are needed to investigate the effects of baicalin on the above RA-related factors among patients, to further understand the pharmarcology of baicalin.

## Conclusion

To conclude, our study hereby supports the clinical efficacy of baicalin in reducing blood lipids and inflammation in patients with both CAD and RA.

## Abbreviations

CAD: coronary artery disease
CT-1: cardiotrophin-1
hs-CRP: high sensitivity C-reactive protein
RA: Patients with rheumatoid arthritis
